# Ethyl­enediammonium tetraaquadi­sulfatomagnesium(II)

**DOI:** 10.1107/S1600536809041981

**Published:** 2009-10-23

**Authors:** Walid Rekik, Houcine Naïli, Tahar Mhiri, Thierry Bataille

**Affiliations:** a Laboratoire de l’Etat Solide, Département de Chimie, Faculté des Sciences de Sfax, BP 802, 3018 SFAX, Tunisia; b Laboratoire de Chimie du Solide et Inorganique Moléculaire (CNRS, UMR 6511), Université de Rennes I, Avenue du Général Leclerc, 35042 Rennes CEDEX, France

## Abstract

The title compound, [NH_3_(CH_2_)_2_NH_3_][Mg(SO_4_)_2_(H_2_O)_4_], was synthesized by the slow evaporation method. Its crystal structure can be described as an alternate stacking of inorganic layers of tetra­aqua­bis(sulfato-*O*)magnesium [Mg(SO_4_)_2_(H_2_O)_4_]^2−^  anions (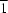 symmetry) and organic layers of [NH_3_(CH_2_)_2_NH_3_]^2+^ cations along the crystallographic *b* axis. The anions, built up from tetrahedral SO_4_ units and octahedral Mg(H_2_O)_4_O_2_ units,  and the cations are linked together through N—H⋯O hydrogen bonds, forming a three-dimensional network. O—H⋯O inter­actions are also present.

## Related literature

For organic–inorganic hybrid solids composed of 3*d* transition metals, sulfate groups and protonated diamines, see: Held (2003 ▶); Naïli *et al.* (2006 ▶); Rekik *et al.* (2005 ▶, 2007 ▶, 2008 ▶, 2009 ▶); Rekik, Naïli, Bataille & Mhiri (2006 ▶); Rekik, Naïli, Bataille *et al.* (2006 ▶); Yahyaoui *et al.* (2007 ▶). For the isostructural manganese, iron and cobalt compounds, see: Chaabouni *et al.* (1996 ▶); Held (2003 ▶); Rekik *et al.* (2008 ▶).
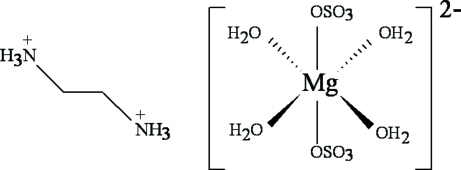


## Experimental

### 

#### Crystal data

(C_2_H_10_N_2_)[Mg(SO_4_)_2_(H_2_O)_4_]
*M* *_r_* = 350.61
Triclinic, 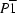

*a* = 6.7847 (4) Å
*b* = 7.0721 (4) Å
*c* = 7.2217 (4) Å
α = 74.909 (2)°
β = 72.378 (2)°
γ = 79.564 (3)°
*V* = 316.89 (3) Å^3^
*Z* = 1
Mo *K*α radiation
μ = 0.53 mm^−1^
*T* = 293 K
0.19 × 0.15 × 0.10 mm

#### Data collection

Nonius KappaCCD diffractometer
Absorption correction: analytical (de Meulenaer & Tompa, 1965 ▶) *T*_min_ = 0.924, *T*_max_ = 0.958
3254 measured reflections
1408 independent reflections
1238 reflections with *I* > 2σ(*I*)
*R*_int_ = 0.099

#### Refinement

*R*[*F* ^2^ > 2σ(*F* ^2^)] = 0.053
*wR*(*F* ^2^) = 0.142
*S* = 1.05
1408 reflections
104 parameters
4 restraints
H atoms treated by a mixture of independent and constrained refinement
Δρ_max_ = 0.69 e Å^−3^
Δρ_min_ = −0.58 e Å^−3^

### 

Data collection: *COLLECT* (Nonius, 1998 ▶); cell refinement: *HKL* *SCALEPACK* (Otwinowski & Minor, 1997 ▶); data reduction: *HKL* *DENZO* (Otwinowski & Minor, 1997 ▶) and *SCALEPACK*; program(s) used to solve structure: *SHELXS97* (Sheldrick, 2008 ▶); program(s) used to refine structure: *SHELXL97* (Sheldrick, 2008 ▶); molecular graphics: *DIAMOND* (Brandenburg & Berndt, 1999 ▶); software used to prepare material for publication: *WinGX* publication routines (Farrugia, 1999 ▶).

## Supplementary Material

Crystal structure: contains datablocks global, I. DOI: 10.1107/S1600536809041981/pb2011sup1.cif

Structure factors: contains datablocks I. DOI: 10.1107/S1600536809041981/pb2011Isup2.hkl

Additional supplementary materials:  crystallographic information; 3D view; checkCIF report

**Fig. 3.**
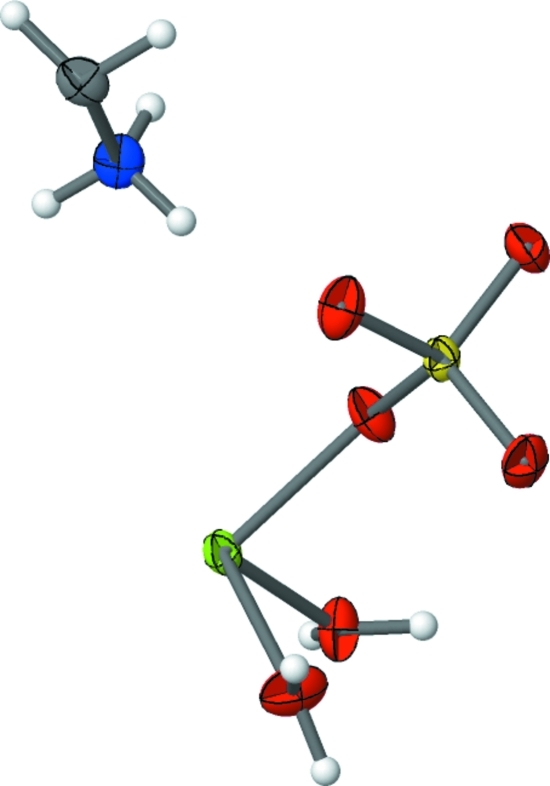
The asymmetric unit of the title compound.

Enhanced figure: interactive version of Fig. 3

## Figures and Tables

**Table 1:** Selected bond distances (Å)

Mg—O*W*1	2.0632 (18)
Mg—O4	2.0826 (15)
Mg—O*W*2	2.0833 (18)
S—O1	1.4605 (17)
S—O2	1.4688 (17)
S—O3	1.4748 (16)
S—O4	1.4844 (15)

**Table 2:** Hydrogen-bond geometry (Å, °)

*D*—H⋯*A*	*D*—H	H⋯*A*	*D*⋯*A*	*D*—H⋯*A*
N—H0*A*⋯O4^iii^	0.89	1.95	2.838 (2)	174
N—H0*B*⋯O3^iv^	0.89	2.05	2.886 (3)	156
N—H0*C*⋯O2	0.89	1.97	2.837 (3)	163
O*W*1—H11⋯O2^v^	0.866 (19)	1.91 (2)	2.767 (2)	169 (4)
O*W*1—H12⋯O3^vi^	0.869 (18)	1.890 (19)	2.758 (3)	178 (3)
O*W*2—H21⋯O1^vii^	0.841 (19)	1.95 (2)	2.729 (2)	153 (3)
O*W*2—H22⋯O1^viii^	0.871 (19)	2.03 (2)	2.869 (2)	162 (4)

Symmetry codes: (iii) 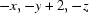; (iv) 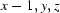; (v) 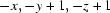; (vi) 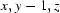; (vii) 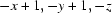; (viii) 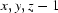.

**Fig. 1.**
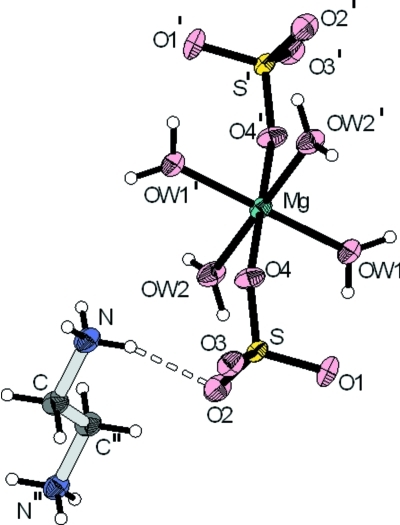
A part of the crystal structure of the title compound showing the asymmetric unit (expanded by symmetry to give complete organic cation and trimeric unit) and atom numbering. Displacement ellipsoids are drawn at the 50% probability level. Hydrogen bonds are represented by dashed lines.[Symmetry codes: (I) -x, -y - 1, -z; (II) -x - 1, -y + 2, -z + 1.]

**Fig. 2.**
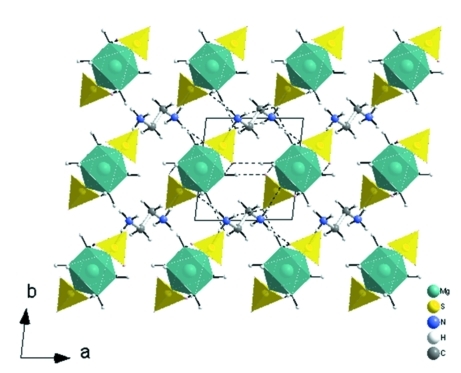
Projection of the crystal structure of the title compound along the c axis, with hydrogen bonds indicated as dashed lines.

## supplementary crystallographic information

### Comment

Organic-inorganic hybrid materials is the subject of major interest, allowing to combines some properties of an inorganic material (or a molecule), and some properties of an organic molecule (or a polymer). This symbiosis between two worlds of chemistry too long regarded as opposites can also lead to completely new properties, and opens a wide field of investigations for the chemist. The applications of these "new" materials cover diverse areas as the properties of strength, optics, ferroelectricity and ferroelasticity, electronics and ionic solid ··· Recently, we reported some new organic-inorganic hybrid solids composed of 3d transition metal, sulfate groups and protonated diamine (Rekik *et al.*, 2005; Naïli, *et al.*, 2006; Rekik *et al.*, 2007; Yahyaoui *et al.*, 2007; Rekik *et al.*, 2008; Rekik *et al.*, 2009). In the field of our investigations in the organic-inorganic hybrid materials, we report here the chemical preparation and the structural characterization of a new magnesium ethylenediammonium bis(sulfate)tetrahydrate,[NH_3_(CH_2_)_2_NH_3_][Mg(SO_4_)_2_(H_2_O)_4_]. The title compound is isostructural with the manganese, iron and cobalt related phases (Chaabouni *et al.*, 1996; Held *et al.*, 2003; Rekik *et al.*, 2008). As it can be seen in figure 1, the asymmetric unit of the title compound contains only one magnesium atom located at a symmetry centre, only one sulfate tetrahedron and ethylenediammonium cation lying about inversion centre. The Mg(II) central atom is octahedrally coordinated by one oxygen atom of sulfate group, two water molecules and the corresponding centrosymmetrically located atoms. Each octahedron around Mg shares two oxygen with two sulfate groups to form trimeric units, [Mg(SO_4_)_2_(H_2_O)_4_]^2-^. The negative charge of the inorganic part is compensated by ethylenediammonium cations which are located on inversion centres in the inorganic framework cavities. The structure cohesion and stability are assured by two types of hydrogen bonds, OW—H···O and N—H···O. Figure 2 shows that the structure can be described as an alternation between organic and inorganic layers along the crystallographic *b* axis.

### Experimental

Single-crystals of the title compound were grown by slow evaporation at room temperature of an aqueous solution of MgSO_4_.7(H_2_O)/C_2_H_8_N_2_ /H_2_SO_4_in a ratio 1:1:1. The product was filtered off and washed with a small amount of distilled water.

### Refinement

The aqua H atoms were located in difference map and refined with O—H distance restraints of 0.85 (2) Å and H—H distance restraints of 1.39 (2) Å. H atoms bonded to C and N atomswere positioned geometrically and allowed to ride on their parent atom, with C—H = 0.97 Å, N—H = 0.89 Å and *U*_iso_ = 1.2*U*_eq_(C, N).

### Crystal data

**Table:**

(C_2_H_10_N_2_)[Mg(SO_4_)_2_(HO)_4_]	*Z* = 1
*M**_r_* = 350.61	*F*(000) = 184
Triclinic, *P*1	*D*_x_ = 1.837 Mg m^−^^3^
Hall symbol: -P 1	Mo *K*α radiation, λ = 0.71073 Å
*a* = 6.7847 (4) Å	Cell parameters from 3254 reflections
*b* = 7.0721 (4) Å	θ = 3.0–27.4°
*c* = 7.2217 (4) Å	µ = 0.53 mm^−^^1^
α = 74.909 (2)°	*T* = 293 K
β = 72.378 (2)°	Prism, colourless
γ = 79.564 (3)°	0.19 × 0.15 × 0.10 mm
*V* = 316.89 (3) Å^3^	

### Data collection

**Table:**

Nonius KappaCCD diffractometer	1408 independent reflections
Radiation source: fine-focus sealed tube	1238 reflections with *I* > 2σ(*I*)
horizonally mounted graphite crystal	*R*_int_ = 0.099
Detector resolution: 9 pixels mm^-1^	θ_max_ = 27.4°, θ_min_ = 3.0°
CCD rotation images, thick slices scans	*h* = −8→8
Absorption correction: analytical (de Meulenaer & Tompa, 1965)	*k* = −9→9
*T*_min_ = 0.924, *T*_max_ = 0.958	*l* = −9→9
3254 measured reflections	

### Refinement

**Table:**

Refinement on *F*^2^	Primary atom site location: structure-invariant direct methods
Least-squares matrix: full	Secondary atom site location: difference Fourier map
*R*[*F*^2^ > 2σ(*F*^2^)] = 0.053	Hydrogen site location: inferred from neighbouring sites
*wR*(*F*^2^) = 0.142	H atoms treated by a mixture of independent and constrained refinement
*S* = 1.05	*w* = 1/[σ^2^(*F*_o_^2^) + (0.0725*P*)^2^ + 0.1042*P*] where *P* = (*F*_o_^2^ + 2*F*_c_^2^)/3
1408 reflections	(Δ/σ)_max_ = 0.001
104 parameters	Δρ_max_ = 0.69 e Å^−^^3^
4 restraints	Δρ_min_ = −0.58 e Å^−^^3^

### Special details

**Table:**

Experimental. Data were corrected for Lorentz-polarization effects and an analytical absorption correction (de Meulenaer & Tompa, 1965) was applied. The structure was solved in the P -1 space group by the direct methods (Mg and S) and subsequent difference Fourier syntheses (all other atoms), with an exception for H atoms bonded to C and N atoms which are positioned geometrically.
Geometry. All e.s.d.'s (except the e.s.d. in the dihedral angle between two l.s. planes) are estimated using the full covariance matrix. The cell e.s.d.'s are taken into account individually in the estimation of e.s.d.'s in distances, angles and torsion angles; correlations between e.s.d.'s in cell parameters are only used when they are defined by crystal symmetry. An approximate (isotropic) treatment of cell e.s.d.'s is used for estimating e.s.d.'s involving l.s. planes.
Refinement. Refinement of *F*^2^ against ALL reflections. The weighted *R*-factor *wR* and goodness of fit *S* are based on *F*^2^, conventional *R*-factors *R* are based on *F*, with *F* set to zero for negative *F*^2^. The threshold expression of *F*^2^ > σ(*F*^2^) is used only for calculating *R*-factors(gt) *etc*. and is not relevant to the choice of reflections for refinement. *R*-factors based on *F*^2^ are statistically about twice as large as those based on *F*, and *R*- factors based on ALL data will be even larger.

### Fractional atomic coordinates and isotropic or equivalent isotropic displacement parameters (Å^2^)

**Table:**

	*x*	*y*	*z*	*U*_iso_*/*U*_eq_	
Mg	0.0000	0.5000	0.0000	0.0186 (3)	
S	0.19880 (7)	0.72309 (7)	0.25068 (7)	0.0178 (3)	
OW1	0.0843 (3)	0.2584 (3)	0.2036 (3)	0.0330 (5)	
OW2	0.2566 (3)	0.4397 (3)	−0.2311 (3)	0.0278 (4)	
O1	0.3220 (3)	0.5558 (3)	0.3453 (3)	0.0282 (4)	
O2	−0.0021 (3)	0.7650 (3)	0.3921 (2)	0.0298 (4)	
O3	0.3142 (3)	0.8975 (2)	0.1783 (2)	0.0258 (4)	
O4	0.1602 (3)	0.6817 (2)	0.0741 (2)	0.0254 (4)	
N	−0.3262 (3)	1.0074 (3)	0.2425 (3)	0.0259 (5)	
H0A	−0.2677	1.1054	0.1482	0.039*	
H0B	−0.4085	0.9564	0.1974	0.039*	
H0C	−0.2274	0.9145	0.2752	0.039*	
C	−0.4508 (4)	1.0834 (3)	0.4201 (3)	0.0246 (5)	
H0D	−0.5582	1.1854	0.3852	0.029*	
H0E	−0.3622	1.1406	0.4691	0.029*	
H11	0.045 (6)	0.262 (6)	0.329 (3)	0.053 (10)*	
H12	0.156 (5)	0.145 (3)	0.193 (5)	0.038 (8)*	
H21	0.378 (3)	0.444 (5)	−0.227 (5)	0.040 (9)*	
H22	0.255 (7)	0.458 (6)	−0.355 (3)	0.058 (11)*	

### Atomic displacement parameters (Å^2^)

**Table:**

	*U*^11^	*U*^22^	*U*^33^	*U*^12^	*U*^13^	*U*^23^
Mg	0.0188 (5)	0.0196 (5)	0.0190 (5)	−0.0045 (4)	−0.0084 (4)	−0.0013 (4)
S	0.0169 (3)	0.0199 (4)	0.0175 (3)	−0.0035 (2)	−0.0079 (2)	−0.0006 (2)
OW1	0.0485 (11)	0.0264 (9)	0.0244 (9)	0.0062 (8)	−0.0186 (8)	−0.0026 (7)
OW2	0.0193 (8)	0.0409 (10)	0.0242 (8)	−0.0047 (7)	−0.0079 (7)	−0.0054 (7)
O1	0.0244 (8)	0.0259 (9)	0.0313 (9)	−0.0002 (7)	−0.0143 (7)	0.0052 (7)
O2	0.0214 (8)	0.0410 (10)	0.0230 (8)	−0.0011 (7)	−0.0043 (7)	−0.0039 (7)
O3	0.0279 (9)	0.0249 (9)	0.0290 (9)	−0.0111 (7)	−0.0134 (7)	−0.0013 (7)
O4	0.0287 (9)	0.0314 (9)	0.0198 (8)	−0.0136 (7)	−0.0086 (7)	−0.0026 (7)
N	0.0257 (10)	0.0291 (10)	0.0204 (9)	−0.0045 (8)	−0.0059 (8)	−0.0005 (8)
C	0.0287 (11)	0.0240 (12)	0.0196 (10)	−0.0073 (9)	−0.0056 (9)	−0.0004 (9)

### Geometric parameters (Å, °)

**Table:**

Mg—OW1	2.0632 (18)	OW1—H12	0.869 (18)
Mg—OW1^i^	2.0632 (18)	OW2—H21	0.841 (19)
Mg—O4^i^	2.0826 (15)	OW2—H22	0.871 (19)
Mg—O4	2.0826 (15)	N—C	1.479 (3)
Mg—OW2	2.0833 (18)	N—H0A	0.8900
Mg—OW2^i^	2.0833 (18)	N—H0B	0.8900
S—O1	1.4605 (17)	N—H0C	0.8900
S—O2	1.4688 (17)	C—C^ii^	1.510 (4)
S—O3	1.4748 (16)	C—H0D	0.9700
S—O4	1.4844 (15)	C—H0E	0.9700
OW1—H11	0.866 (19)		
			
OW1—Mg—OW1^i^	180.00 (13)	O3—S—O4	106.73 (9)
OW1—Mg—O4^i^	87.87 (7)	Mg—OW1—H11	119 (3)
OW1^i^—Mg—O4^i^	92.13 (7)	Mg—OW1—H12	133 (2)
OW1—Mg—O4	92.13 (7)	H11—OW1—H12	108 (4)
OW1^i^—Mg—O4	87.87 (7)	Mg—OW2—H21	121 (2)
O4^i^—Mg—O4	180.000 (1)	Mg—OW2—H22	125 (3)
OW1—Mg—OW2	93.30 (8)	H21—OW2—H22	109 (4)
OW1^i^—Mg—OW2	86.70 (8)	S—O4—Mg	140.66 (9)
O4^i^—Mg—OW2	88.50 (7)	C—N—H0A	109.5
O4—Mg—OW2	91.50 (7)	C—N—H0B	109.5
OW1—Mg—OW2^i^	86.70 (8)	H0A—N—H0B	109.5
OW1^i^—Mg—OW2^i^	93.30 (8)	C—N—H0C	109.5
O4^i^—Mg—OW2^i^	91.50 (7)	H0A—N—H0C	109.5
O4—Mg—OW2^i^	88.50 (7)	H0B—N—H0C	109.5
OW2—Mg—OW2^i^	180.00 (8)	N—C—C^ii^	109.3 (2)
O1—S—O2	110.22 (10)	N—C—H0D	109.8
O1—S—O3	109.88 (10)	C^ii^—C—H0D	109.8
O2—S—O3	110.33 (11)	N—C—H0E	109.8
O1—S—O4	110.76 (10)	C^ii^—C—H0E	109.8
O2—S—O4	108.85 (9)	H0D—C—H0E	108.3

Symmetry codes: (i) −*x*, −*y*+1, −*z*; (ii) −*x*−1, −*y*+2, −*z*+1.

### Hydrogen-bond geometry (Å, °)

**Table:**

*D*—H···*A*	*D*—H	H···*A*	*D*···*A*	*D*—H···*A*
N—H0A···O4^iii^	0.89	1.95	2.838 (2)	174
N—H0B···O3^iv^	0.89	2.05	2.886 (3)	156
N—H0C···O2	0.89	1.97	2.837 (3)	163
OW1—H11···O2^v^	0.87 (2)	1.91 (2)	2.767 (2)	169 (4)
OW1—H12···O3^vi^	0.87 (2)	1.89 (2)	2.758 (3)	178 (3)
OW2—H21···O1^vii^	0.84 (2)	1.95 (2)	2.729 (2)	153 (3)
OW2—H22···O1^viii^	0.87 (2)	2.03 (2)	2.869 (2)	162 (4)

Symmetry codes: (iii) −*x*, −*y*+2, −*z*; (iv) *x*−1, *y*, *z*; (v) −*x*, −*y*+1, −*z*+1; (vi) *x*, *y*−1, *z*; (vii) −*x*+1, −*y*+1, −*z*; (viii) *x*, *y*, *z*−1.

## References

[bb1] Brandenburg, K. & Berndt, M. (1999). *DIAMOND* Crystal Impact GbR, Bonn, Germany.

[bb2] Chaabouni, S., Kamoun, S., Daoud, A. & Jouini, T. (1996). *Acta Cryst.* C**52**, 505–506.

[bb3] Farrugia, L. J. (1999). *J. Appl. Cryst.* **32**, 837–838.

[bb4] Held, P. (2003). *Acta Cryst.* E**59**, m197–m198.

[bb5] Meulenaer, J. de & Tompa, H. (1965). *Acta Cryst.* **19**, 1014–1018.

[bb6] Naïli, H., Rekik, W., Bataille, T. & Mhiri, T. (2006). *Polyhedron*, **25**, 3543–3554.

[bb7] Nonius (1998). *COLLECT* Nonius BV, Delft, The Netherlands.

[bb8] Otwinowski, Z. & Minor, W. (1997). *Methods in Enzymology*, Vol. 276, *Macromolecular Crystallography*, Part A, edited by C. W. Carter Jr & R. M. Sweet, pp 307–326. New York: Academic Press.

[bb9] Rekik, W., Naïli, H., Bataille, T. & Mhiri, T. (2006). *J. Organomet. Chem.* **691**, 4725–4732.

[bb10] Rekik, W., Naïli, H., Bataille, T., Roisnel, T. & Mhiri, T. (2006). *Inorg. Chim. Acta*, **359**, 3954–3962.

[bb11] Rekik, W., Naïli, H., Mhiri, T. & Bataille, T. (2005). *Acta Cryst.* E**61**, m629–m631.

[bb12] Rekik, W., Naïli, H., Mhiri, T. & Bataille, T. (2007). *J. Chem. Crystallogr.* **37**, 147–155.

[bb13] Rekik, W., Naïli, H., Mhiri, T. & Bataille, T. (2008). *Mater. Res. Bull.*, **43**, 2709–2718.

[bb14] Rekik, W., Naïli, H., Mhiri, T. & Bataille, T. (2009). *J. Solid State Sci.* **11**, 614–621.

[bb15] Sheldrick, G. M. (2008). *Acta Cryst.* A**64**, 112–122. 10.1107/S0108767307043930 18156677

[bb16] Yahyaoui, S., Rekik, W., Naïli, H., Mhiri, T. & Bataille, T. (2007). *J. Solid State Chem.* **180**, 3560–3570.

